# A Constitutive Expression System for Cellulase Secretion in *Escherichia coli* and Its Use in Bioethanol Production

**DOI:** 10.1371/journal.pone.0119917

**Published:** 2015-03-13

**Authors:** Neha Munjal, Kamran Jawed, Saima Wajid, Syed Shams Yazdani

**Affiliations:** 1 Synthetic Biology and Biofuels Group, International Centre for Genetic Engineering and Biotechnology, Aruna Asaf Ali Marg, New Delhi, India; 2 DBT-ICGEB Centre for Advanced Bioenergy Research, International Centre for Genetic Engineering and Biotechnology, Aruna Asaf Ali Marg, New Delhi, India; 3 Centre for Biotechnology, Jamia Hamdard, Hamdard Nagar, New Delhi, India; Korea University, KOREA, REPUBLIC OF

## Abstract

The production of biofuels from lignocellulosic biomass appears to be attractive and viable due to the abundance and availability of this biomass. The hydrolysis of this biomass, however, is challenging because of the complex lignocellulosic structure. The ability to produce hydrolytic cellulase enzymes in a cost-effective manner will certainly accelerate the process of making lignocellulosic ethanol production a commercial reality. These cellulases may need to be produced aerobically to generate large amounts of protein in a short time or anaerobically to produce biofuels from cellulose via consolidated bioprocessing. Therefore, it is important to identify a promoter that can constitutively drive the expression of cellulases under both aerobic and anaerobic conditions without the need for an inducer. Using *lacZ* as reporter gene, we analyzed the strength of the promoters of four genes, namely *lacZ*, *gapA*, *ldhA* and *pflB*, and found that the *gapA* promoter yielded the maximum expression of the β-galactosidase enzyme under both aerobic and anaerobic conditions. We further cloned the genes for two cellulolytic enzymes, β-1,4-endoglucanase and β-1,4-glucosidase, under the control of the *gapA* promoter, and we expressed these genes in *Escherichia coli*, which secreted the products into the extracellular medium. An ethanologenic *E*. *coli*strain transformed with the secretory β-glucosidase gene construct fermented cellobiose in both defined and complex medium. This recombinant strain also fermented wheat straw hydrolysate containing glucose, xylose and cellobiose into ethanol with an 85% efficiency of biotransformation. An ethanologenic strain that constitutively secretes a cellulolytic enzyme is a promising platform for producing lignocellulosic ethanol.

## Introduction

The abundance of lignocellulosic biomass and its renewable nature makes it an ideal feedstock for biofuel production [1, 2]. Several enzymes have been reported in the literature that hydrolyze the cellulose and hemicellulose portion of the lignocellulose, but due to the complex nature of the material, a cocktail of enzymes is used to hydrolyze the biomass into fermentable monomeric sugars [3]. The four key enzymes responsible for the degradation of cellulose are endoglucanase, exoglucanase, β-glucosidase and polysaccharide monooxygenase, while hemicellulose is mostly degraded by xylanase and β-xylosidase [4, 5].

The production cost of cellulolytic enzymes has declined over the last decade [6], but it is still high enough to seriously affect the lignocellulosic ethanol process [7]. Most of the cellulolytic enzymes currently available in the market are from fungal sources; however, bacterial cellulases have recently received attention due to their higher specific activity, thermostability and robust pH range [8]. Several bacterial enzymes have been characterized and overexpressed in heterologous hosts [9, 10]. Often commercially available inducible expression systems based on T5 and T7 promoters are used for overexpression of enzymes in the heterologous hosts [11, 12]. These promoters are induced either by adding chemicals or by giving the heat—shock [13]. Use of chemicals adds to the production cost and also leads to the toxicity to the host cells. Giving heat shock, on the other hand, leads to induction of heat-shock responses and up-regulation of cellular proteases. Therefore, expression of recombinant enzymes under the control of a constitutive promoter will minimize these issues. A constitutive promoter that works efficiently under both aerobic and anaerobic condition will be even more useful. Microbial growth in aerobic conditions is typically used to achieve a large amount of biomass and a high level of protein expression [14, 15]. In contrast, microbial growth in anaerobic condition is used to produce several biofuel molecules [16], and expression of cellulolytic enzymes under this condition could either lead to a reduced enzyme load for hydrolysis during simultaneous saccharification and fermentation (SSF) [17, 18] or completely circumvent the use of hydrolytic enzymes via consolidated bioprocessing (CBP) [19, 20]. The expression of enzymes such as β-glucosidase under anaerobic conditions will also help in the hydrolysis and consumption of partially hydrolyzed soluble cellulosic materials, such as cellobiose and cello-oligosaccharides [21]. For this to occur, the cellobiose either needs to be transported inside the cells through various transporters or the enzyme must be secreted to the extracellular medium to hydrolyze its substrate. A fungal transporter has been expressed in *Saccharomyces cerevisiae* for the import of cellobiose and for its hydrolysis and fermentation [22, 23]. Similarly, a β-glucosidase from *Paenibacillus polymyxa* has been expressed in *E*. *coli* and secreted into the extracellular medium with the help of an OsmY (a hyperosmotically inducible periplasmic protein) tag for the hydrolysis of cellobiose [24].

In this study, we have screened a constitutive promoter with the help of the *lacZ* reporter gene to generate protein expression under both aerobic and anaerobic conditions. We have cloned the genes for endoglucanase and β-glucosidase under the control of a constitutive promoter and compared their expression kinetics to those for the expression with an inducible promoter. We have further demonstrated that the β-glucosidase secreted by an ethanologenic strain can hydrolyze cellobiose and facilitate its fermentation. Moreover, we show that a biomass hydrolysate composed of glucose, xylose and cellobiose can be fermented successfully with a high ethanol yield by the recombinant ethanologenic strain.

## Materials and Methods

### Media and reagents

Bacterial cultures were grown in LB medium with kanamycin (30 μg/ml) or ampicillin (100 μg/ml), as applicable. All media components were purchased from HiMedia Laboratories while the antibiotics were purchased from Sigma-Aldrich. Restrictions enzymes and T4 DNA ligase were purchased from New England Biolabs to perform various molecular biology experiments. Phusion High Fidelity DNA polymerase (Finnzymes) was used for template amplification for cloning purposes, and Taq polymerase (Bangalore Genei) was used for PCR for validation purposes. The primers for all PCR amplifications were synthesized by Sigma-Aldrich.

### Bacterial strains and plasmid construction

The bacterial strains, plasmids and primers used in this study are shown in Table 1. We selected the wild-type *E*. *coli* B strain to perform all the experiments because derivative of this strain has been found to be a good ethanol producer [25, 26], and another derivative, BL21(DE3), is widely used for recombinant protein production [27]. To identify suitable promoters that can express cellulase enzymes constitutively under both aerobic and anaerobic conditions, we replaced the native promoter for the *lacZ* gene in the *E*. *coli* B genome with the promoters for the *gapA*, *ldhA* and *pflB* genes of *E*. *coli* B, as described earlier [25]. Briefly, the FRT-kan-FRT nucleotide cassette was amplified from the pKD4 plasmid and cloned at the *Eco*RI/*Bam*HI sites of the pUC19 vector to obtain a pSSY01 plasmid. The promoter and the ribosomal binding site of the *gapA*, *ldhA* and *pflB* genes were amplified from the *E*. *coli* B genome and ligated downstream of FRT-kan-FRT at the *Bam*HI/*Hind*III sites of pSSY01 to generate the pSSY02, pSSY04 and pSSY06 plasmids, respectively (Table 1). A 45-base sequence corresponding to the-28 to +17 nucleotide position with respect to the *lacI* coding region was added to the 20 bases of the 5’ end of the FRT-kan-FRT sequence to generate the H1 primer, and a 45-base sequence corresponding to the +1 to +45 position of the *lacZ* coding region was added to the 20–22 bases of the 3’ end of each heterologous promoter to obtain the H2 primer (Table 1). PCR was performed with the H1/H2 primers using the pSSY02, pSSY04 and pSSY06 plasmids as a template under following conditions- 98°C for 2 min, followed by 30 cycles of denaturation at 98°C for 15 sec, annealing at 59°C for 15 sec, extension at 72°C for 2 min and a final extension at 72°C for 10 min. The PCR product was gel eluted, *Dpn*I digested and electroporated in *E*. *coli* B cells carrying the pKD46 plasmid to obtain SSYL1, SSYL2 and SSYL3 strains for *gapA*, *ldhA* and *pflB* promoters, respectively. Transformants were selected on LB-agar plates containing 30 μg/ml kanamycin and verified by colony PCR for promoter replacement using V_lacZ_F and V_lacZ_R primers (Table 1).

**Table 1:** Strains, plasmids and primers used in the study.

Name	Description	Reference
**Strains**		
*E*. *coli* B	F-	CGSC #2507
*E*. *coli* BLR(DE3)	*F—ompT hsdS*_*B*_ *(r*_*B*_^-^ *m*_*B*_^*-*^ *) gal lac dcm (DE3) Δ(srl- recA)306*::*Tn10(Tet* ^*R*^)	Novagen
SSYL1	*E*. *coli* B, ΔlacZ-promoter::*FRT-kan-FRT-gapA* gene promoter; promoter of *lacZ* gene replaced with promoter of *gapA* gene	This study
SSYL2	*E*. *coli* B, ΔlacZ-promoter::*FRT-kan-FRT-ldhA* gene promoter; promoter of *lacZ* gene replaced with promoter of *ldhA* gene	This study
SSYL3	*E*. *coli* B, ΔlacZ-promoter::*FRT-kan-FRT-pflB* gene promoter; promoter of *lacZ* gene replaced with promoter of *pflB* gene	This study
SSY09	*E*. *coli* B, ΔPDH-promoter:: *gapA* gene promoter, *ΔldhA*, *ΔfrdA*, *ΔackA*, *ΔpflB*::*FRT-kan-FRT*	[22]
SSY11	*E*. *coli* B, ΔPDH-promoter:: *gapA* gene promoter, *ΔldhA*, *ΔfrdA*, *ΔackA*, *ΔpflB*	This study
SSY12	SSY11 with pPgap-OsmY-Gluc1C and pZSack plasmid	This study
**Plasmids**		
pKD46	*bla*, *γ β exo* (red recombinase), temperature-conditional replicon	CGSC #7739
pCP20	*bla*, *flp*, temperature-conditional replicon	CGSC #7629
pSSY01	FRT-kan-FRT sequence from pKD4 was cloned into pUC19 at *Eco*RI and *Bam*HI sites	[22]
pSSY02	*gapA* gene promoter from *E*. *coli* B was cloned into pSSY01 at *Bam*HI and *Hind*III sites	[22]
pSSY04	*ldhA* gene promoter from *E*. *coli* B was cloned into pSSY01 at *Bam*HI and *Hind*III sites	[22]
pSSY06	*pflB* gene promoter from *E*. *coli* B was cloned into pSSY01 at *Bam*HI and *Hind*III sites	[22]
pZSack	*ack* gene cloned in pZS*mcs vector	[22]
pET-PlacZ-lacZ	T7 Promoter of pET28a(+) vector replaced with *lacZ* promoter to express *lacZ*	This Study
pET-PgapA-lacZ	T7 Promoter of pET28a(+) vector replaced with *gapA* promoter to express *lacZ*	This Study
pET-PldhA-lacZ	T7 Promoter of pET28a(+) vector replaced with *ldhA* promoter to express *lacZ*	This Study
pET-PpflB-lacZ	T7 Promoter of pET28a(+) vector replaced with *pflB* promoter to express *lacZ*	This Study
pET-OsmY-Endo5A	endoglucanase gene fused with *osmY* gene at 5’-end under T7 promoter	[21]
pET–OsmY–Gluc1C	β-glucosidase gene fused with *osmY* gene at 5’-end under T7 promoter	[21]
pPgap-OsmY-Endo5A	T7 promoter of pET-OsmY-Endo5A vector replaced with *gapA* promoter	This study
pPgap-OsmY-Gluc1C	T7 Promoter of pET–OsmY–Gluc1C vector replaced with *gapA* promoter	This study
**Primers**		
H1_lacZ	*GGAAGAGAGTCAATTCAGGGTGGTGAATGTGAAACCAGTAACGTT*GTGTAGGCTGGAGCTGCTTC	This study
H2_lacZ_PldhA	*ACGACGTTGTAAAACGACGGCCAGTGAATCCGTAATCATGGTCAT*AAGACTTTCTCCAGTGATGTTG	This study
H2_lacZ_PgapA	*ACGACGTTGTAAAACGACGGCCAGTGAATCCGTAATCATGGTCAT*ATATTCCACCAGCTATTTGT	This study
H2_lacZ_PpflB	*ACGACGTTGTAAAACGACGGCCAGTGAATCCGTAATCATGGTCAT* GTAACACCTACCTTCTGTTGC TGTGATATAGAAGAC	This study
V_lacZ_F	TATCGGCCTCAGGAAGATCGC	This study
V_lacZ_R	GTGAATGTGAAACCAGTAACG	This study
PlacZ_lacZ_F	GAAGATCTCAATACGCAAACCGCCTCTC	This study
PgapA_lacZ_F	GAAGATCTGATTCTAACAAAACATTAACAC	This study
PldhA_lacZ_F	GAAGATCTGCAAGCTGACAATCTCCC	This study
PpflB_lacZ_F	GAAGATCTAACCATGCGAGTTACGGGCCTATAA	This study
lacZ_R	ACGGTCGACTTATTTTTGACACCAGACCAACTG	This study
pET28_Pgap_F	GAAGATCTGATTCTAACAAAACATTAACAC	This study
pET28_Pgap_R	CATGCCATGGATATTCCACCAGCTATTTGT	This study

Note: the enzyme sites are underlined and homologous regions are in italic.

To check β-galactosidase activity at plasmid level, we cloned promoters for *lacZ*, *gapA*, *ldhA* and *pflB* genes upstream of *lacZ* gene in pET28a(+) vector after replacing the T7 promoter. PCR was performed with the primers PlacZ_lacZ_F/lacZ_R, PgapA_lacZ_F/lacZ_R, PldhA_lacZ_F/lacZ_R, PpflB_lacZ_F/lacZ_R (Table 1) using the template as *E*. *coli* B, SSYL1, SSYL2 and SSYL3 genomic DNA to amplify Plac-lacZ, PgapA-lacZ, PldhA-lacZ and PpflB-lacZ regions, respectively, under following conditions- 98°C for 2 min, followed by 30 cycles of denaturation at 98°C for 15 sec, annealing at (54–65)°C for 30 sec, extension at 72°C for 2 min and a final extension at 72°C for 10 min. The PCR products were gel eluted, digested with *Bgl*II and *Sal*I and and ligated to the corresponding restriction sites of pET28a(+) vector and transformed in *E*. *coli* BLR(DE3) for expression.

For the constitutive expression of cellulase enzymes, two plasmids were selected from our previous study [24], pET-OsmY-Endo5A and pET-OsmY-Gluc1C, for the extracellular expression of an endoglucanase and a β-glucosidase, respectively. The T7 promoter of these plasmids was replaced with the promoter for the of *E*. *coli gapA* gene by amplifying the *gapA* promoter from the genome of *E*. *coli* B with the help of the pET28_Pgap_F and pET28_Pgap_R primers listed in Table 1 and cloning at the *Bgl*II and *Nco*I restriction sites of pET-OsmY-Endo5A and pET-OsmY-Gluc1C to obtain the pPgap-OsmY-Endo5A and pPgap-OsmY-Gluc1C plasmids, respectively. These plasmids were used to transform *E*. *coli* BLR(DE3) for checking the enzyme expression.

For expressing β-glucosidase in an ethanologenic strain, we considered SSY09 strain from our previous work as the background host strain [25]. The kanamycin resistance marker was removed from the genome of SSY09 strain with the help of FLP recombinase enzyme supplied through the pCP20 plasmid to obtain SSY11 strain. The SSY11 was used as host to co-transform pPgap-OsmY-Gluc1C plasmid for β-glucosidase expression and pZSack plasmid to enhance growth under microaerobic condition [25] to obtain the final strain SSY12 (Table 1).

### Enzyme assay

For the β-galactosidase assay, cells were grown aerobically in 5 ml of LB medium containing 0.1 mM IPTG in culture tubes for 16 hr and were also grown anaerobically in sealed Hungate tubes filled with 13.5 ml of LB medium for 24 hr. The grown cells were harvested by centrifugation, and the cell pellet was used to assay the β-galactosidase activity. The assay for the *lacZ*-encoded β-galactosidase enzyme was performed as described earlier [28]. Briefly, equal volumes of culture broth and Z buffer (60 mM Na_2_HPO_4_.7H_2_O, 40 mM NaH_2_PO_4_.H_2_O, 10 mM KCl, 1 mM MgSO_4_.7H_2_O, and 50 mM β-mercaptoethanol) were mixed, and the cells were permeabilized by adding one drop of 0.1% SDS and 2 drops of chloroform. The reaction was initiated by adding 0.2 ml of O-nitrophenyl-β-galactoside (ONPG) (4 mg/ml) and the mixture was incubated at 30°C for 10 min. The reaction was terminated by the addition of 1 ml of 1 M Na_2_CO_3_ and the absorbance at 420 nm and 550 nm was measured. The activities were expressed in Miller units and calculated using the following formula: Miller unit = 1000 x [(OD_420_–1.75 x OD_550_)] / (T x V x OD_600_), where T is the time of incubation and V is the reaction volume.

Assays for endoglucanase and β-glucosidase were performed as follows. *E*. *coli* BLR(DE3) cells transformed with the pPgap- and pET-based plasmids were grown aerobically in 5 ml of LB medium containing antibiotics in culture tubes for 16 hr. The culture medium for transformants containing pET-based plasmids also contained 0.1 mM IPTG as inducer. To measure the kinetics of cell growth and enzyme activity under anaerobic condition, the cultures were grown in 50 ml and harvested at various time intervals. The supernatant and cell pellet were used to assay the endoglucanase and β-glucosidase enzymes using the 3,5-dinitrosalicylic acid (DNSA) and para-nitrophenol methods, respectively, as mentioned earlier [24]. One unit of endoglucanase or β-glucosidase activity was defined as the amount of enzyme that released 1 μmol of reducing sugar or p-nitrophenol, respectively, per minute from the substrate. All experiments for the enzyme assays were performed in duplicate, and the data are presented as the average and standard deviation of two independent experiments.

### Cultivation in the bioreactor

For batch fermentation with cellobiose as carbon source, SSY12 cells were seed-cultured in 50 ml of LB medium overnight in tightly sealed serum bottles, harvested and resuspended in fresh medium. The cells were inoculated in a multi-vessel bioreactor (Sartorius Biostat Q plus) containing 350 ml of LB or MOPS (Morpholino-propanesulfonic acid) minimal medium [29] along with 8 g/L cellobiose. The temperature and pH of the culture were maintained at 37°C and 6.3, respectively, using a PID controller. Mixing was achieved using a constant stirring speed of 300 rpm. Microaerobic conditions were established by passing compressed air though the vessel headspace at the rate of 0.02 L/min; in this condition, a dissolved oxygen probe showed a zero reading throughout the fermentation. Samples were withdrawn at regular intervals for cell growth and metabolite measurements.

Fermentation of the biomass hydrolysate was performed as follows. Ammonium-treated wheat straw, a kind gift from Dr. Arvind Lali (Lali et al., 2010, patent application PCT/IN2010/000634), was hydrolyzed using 100 FPU/g dry weight of a cellulase enzyme cocktail (Advanced Enzyme Technology Ltd) in 50 mM citrate buffer (pH 4.8) at 50°C. The clear supernatant was transferred to a fresh bottle and was heat inactivated at 100°C for 20 min and chilled immediately by placing it on ice. This process helped to denature and precipitate the cellulase enzyme, which was then separated by centrifugation at 8000 rpm for 20 min. The clear hydrolysate (200 ml) was filter-sterilized and added to 150 ml of 2.3xLB medium in a 0.5 L capacity multi-vessel bioreactor. The fermentation of the hydrolysate was performed using the SSY12 strain as mentioned above. All fermentations were performed in duplicate, and the data in the figures represent the average of two bioreactor batches.

### Analytical methods

The concentrations of glucose, xylose, cellobiose, ethanol and other metabolites were measured using an HPLC instrument (Agilent) equipped with an RI detector. An Aminex HPX-87H anion exchange column (Bio-Rad) maintained at 40°C was used for sample separation. Filtered and degassed 4 mM H_2_SO_4_ was used as the mobile phase at a constant rate of 0.3 ml/min. Standards of the metabolites (Absolute Standards) at 1 g/L were separated on an HPLC column, and the obtained areas were used to calculate the metabolite concentration in the test samples. The cell growth during cultivation was measured in a spectrophotometer (GE Healthcare) at 600 nm.

## Results

### Identification of a strong constitutive promoter

**Fig 1:**
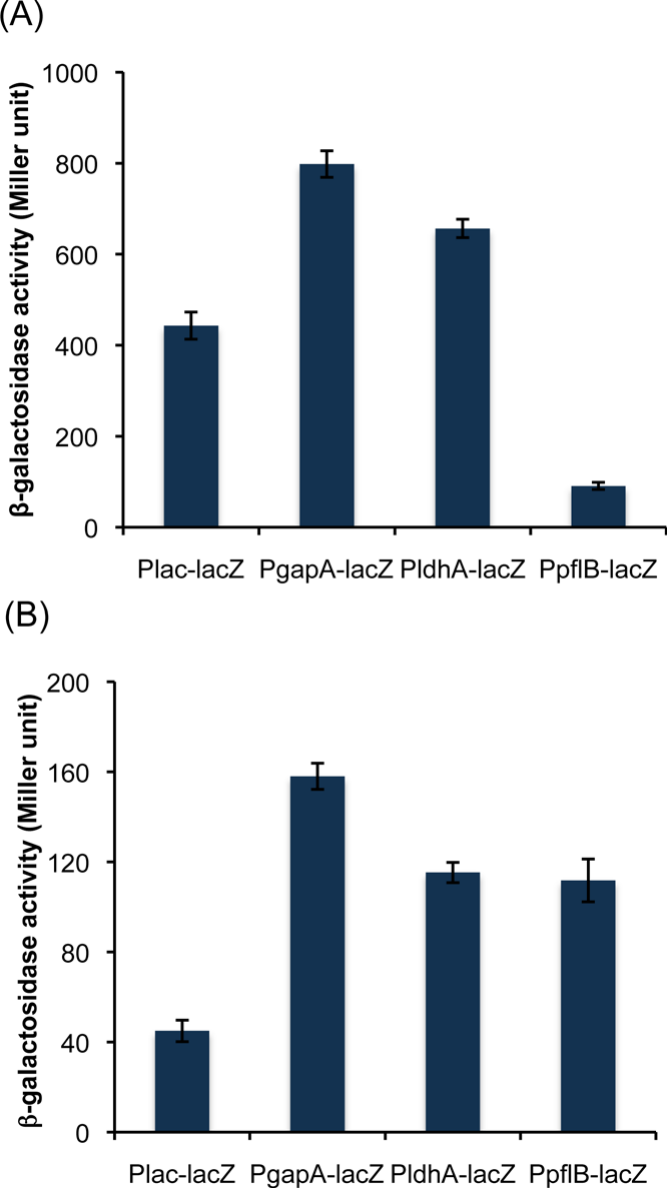
Expression of β-galactosidase via its native and heterologous promoter in genome integration based system. Cells were grown (A) aerobically and (B) anaerobically, harvested and used to monitor β-galactosidase activity. The data are presented as the average and standard deviation of two independent experiments.

**Fig 2:**
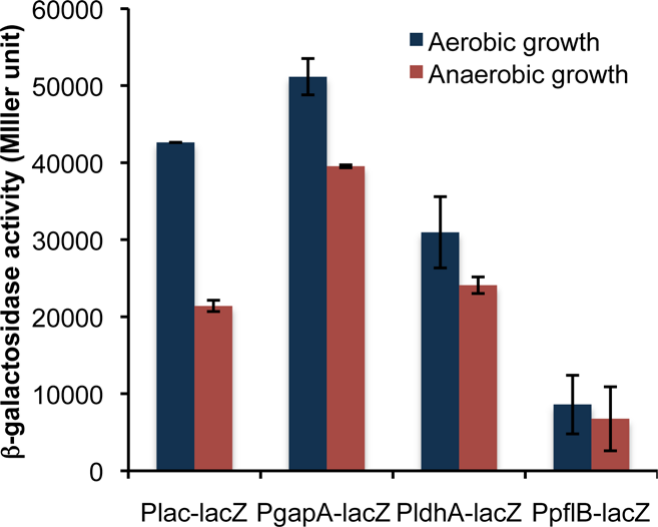
Expression of β-galactosidase via its native and heterologous promoter in plasmid based system. Cells were grown aerobically and anaerobically, harvested and used to monitor β-galactosidase activity. The data are presented as the average and standard deviation of two independent experiments.

We selected three genes, i.e., *glyceraldehyde-3-phosphate dehydrogenase (gapA)*, *lactate dehydrogenase (ldhA) and pyruvate-formate lyase (pflB)*, and tested the strength of their promoters by integrating them into the bacterial genome upstream of a reporter gene, *lacZ*. The native promoter and the RBS of *lacZ* were replaced with the test promoters and their corresponding RBSs. The promoter strengths were studied using the *lacZ* expression under both aerobic and anaerobic conditions. The expression of *lacZ* under the control of its native promoter was tested in the presence of IPTG under a de-repressed condition and was found to be 443 Miller units under the aerobic growth condition (Fig. 1A). The expression of *lacZ* while under the control of the *gapA*, *ldhA* and *pflB* promoters was 1.8-fold higher, 1.5-fold higher and 4.9-fold lower, respectively, compared with that of the native *lacZ* promoter (Fig. 1A). Under the anaerobic condition, the *gapA*, *ldhA* and *pflB* promoters showed 3.5-, 2.6- and 2.5-fold higher expression of *lacZ*, respectively, compared with that of the native *lacZ* promoter (Fig. 1B). To validate the findings in a plasmid-based system, we cloned the *lacZ* gene in pET28a(+) vector under the control of its native promoter as well as under the promoters for *gapA*, *ldhA* and *pflB* genes. All the promoters showed enhanced expression of β-galactosidase in the plasmid-based system as compared to genome integration-based system (Fig. 2). Because the *gapA* promoter yielded the highest expression of *lacZ* under both the aerobic and anaerobic condition, it was selected for further study.

### Constitutive expression of β-1,4-endoglucanase and β-1,4-glucosidase

**Fig 3:**
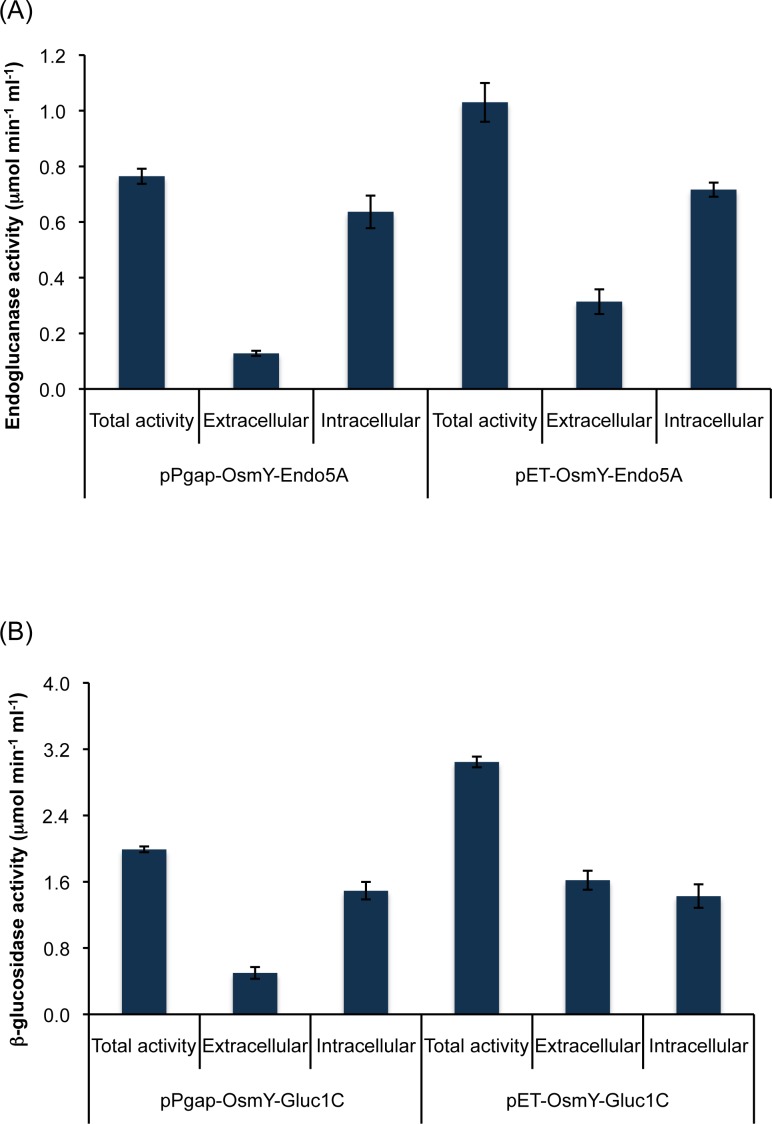
Expression of cellulases under the constitutive *gapA* promoter and the inducible T7 promoter. Cells were grown aerobically for 16 hr, harvested and used to monitor the endoglucanase (A) and β-glucosidase (B) and activity in both the extracellular and intracellular fractions. The data are presented as the average and standard deviation of two independent experiments.

In our previous study, we used a T7 promoter-based expression system, pET28a(+), for the secretion of endoglucanase and β-glucosidase [24]. Here, we replaced the T7 promoter of this vector with the constitutive *gapA* promoter as mentioned in Materials and Methods section, and analyzed the expression and secretion of the enzymes that were aerobically grown in *E*. *coli* BLR(DE3) cells. The total activities of the endoglucanase and β-glucosidase that were expressed under the *gapA* promoter were 0.76 and 2 μmol min^-1^ ml^-1^, the values that were 1.3 and 1.5-fold lower than those under the T7 promoter, respectively (Fig. 3A and 3B). We also found that these enzymes were secreted in the extracellular medium, and the extent of secretion was higher for β-glucosidase than for endoglucanase (Fig. 3A and 3B).

**Fig 4:**
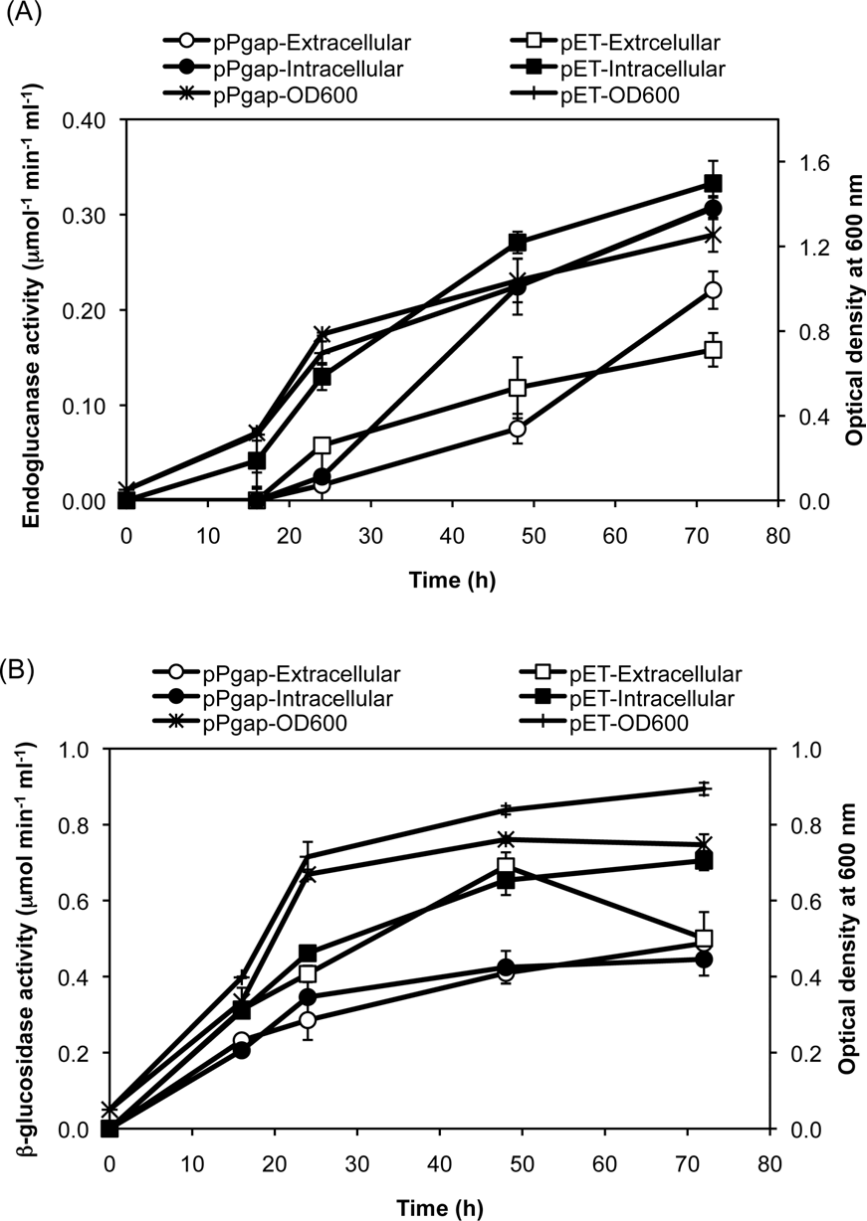
Time profiles of anaerobic cellulase expression under the constitutive *gapA* promoter and the inducible T7 promoter. Cells were grown anaerobically and used to monitor the (A) endoglucanase and (B) β-glucosidase activity in both the extracellular and intracellular fractions. The data are presented as the average and standard deviation of two independent experiments.

We further tested expression of the endoglucanase and β-glucosidase under the anaerobic condition for their use in SSF or CBP. Since expression of cellulolytic enzymes under anaerobic condition will need to be sustainable for longer duration of time to match with biofuel productivity of the host [25], we tested the expression of the enzymes at different time points under anaerobic condition. The time kinetics data revealed that the extracellular expression of endoglucanase under the control of the *gapA* promoter was lower during the early period of growth, but reached a higher level in the later phase. The intracellular endoglucanase level maintained a steady increase for both types of promoters (Fig. 4A). In the case of β-glucosidase, a maximum jump in activity was observed in first 24 hr (Fig. 4B). There was a marginal increase in the β-glucosidase activity in all the samples after 24 hr, except for the extracellular enzyme activity of the T7 based promoter, which declined during the last 24 hr of growth (Fig. 4B). These growth and activity patterns resulted in higher specific activities for endoglucanase and β-glucosidase at 72 hr as compared to 24 hr under the anaerobic condition (Table 2).

**Table 2:** Enzyme activity of endoglucanase and β-glucosidase under aerobic and anaerobic cultivation.

Growth Conditions				Endoglucanase activity		β-glucosidase activity	
				Volumetric activity (μmol min^-1^ ml^-1^)	Specific activity (μmol min^-1^ OD_600_^-1^)	Volumetric activity (μmol min^-1^ ml^-1^)	Specific activity (μmol min^-1^ OD_600_^-1^)
**Aerobic Growth** ^a^	Extracellular	*gapA* promoter	At 16 hr	0.13	0.06	0.50	0.28
		T7 promoter	At 16 hr	0.31	0.15	1.6	0.86
	Intracellular	*gapA* promoter	At 16 hr	0.64	0.28	1.49	0.84
		T7 promoter	At 16 hr	0.72	0.35	1.42	0.76
**Anaerobic Growth** ^b^	Extracellular	*gapA* promoter	At 24 hr	0.02	0.02	0.29	0.43
			At 72 hr	0.22	0.18	0.49	0.65
		T7 promoter	At 24 hr	0.06	0.08	0.41	0.57
			At 72 hr	0.16	0.11	0.50	0.56
	Intracellular	*gapA* promoter	At 24 hr	0.03	0.03	0.35	0.52
			At 72 hr	0.31	0.24	0.45	0.60
		T7 promoter	At 24 hr	0.13	0.19	0.46	0.65
			At 72 hr	0.33	0.24	0.71	0.79

^a^Aerobic growth data is based on single time point study in 5 ml culture volume

^b^Anaerobic growth data is based on time kinetic study in 50 ml culture volume

### Fermentation of cellobiose by the engineered strain

**Fig 5:**
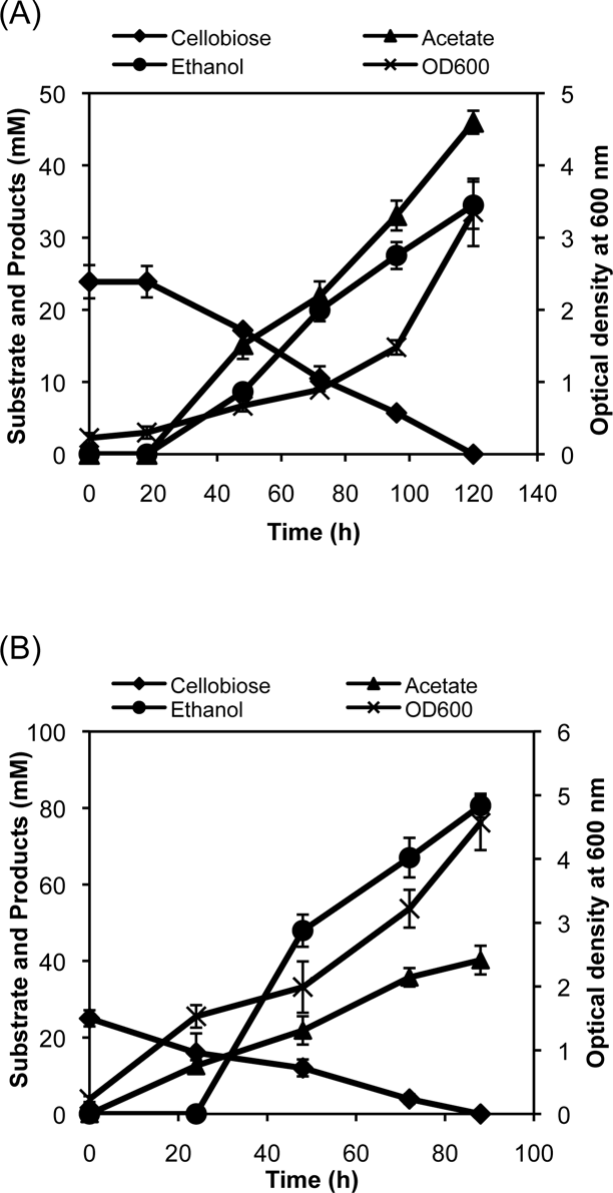
Time profiles of cellobiose fermentation by the engineered strain. The engineered *E*. *coli* strain SSY12 bearing the plasmid pPgap-OsmY-Gluc1C was grown in minimal medium (A) or complex medium (B) containing cellobiose under a microaerobic condition, and the metabolites and cell growth were monitored throughout the cultivation period.

We showed in our earlier report that the β-glucosidase expressed in this study under the control of a constitutive promoter can hydrolyze cellobiose and the cello-oligosaccharides that have a chain length of up to five glucose [10]. In our earlier study, we also constructed an ethanologenic strain via modulation of the endogenous pathway for the fermentation of glucose and xylose into ethanol [25]. We wanted to determine whether this ethanologenic strain SSY09(pZSack) (Table 1) could ferment cellobiose after the secretion of β-glucosidase via the plasmid pPgap-OsmY-Gluc1C. The FRT-kan-FRT cassette from the genome of this strain was removed because it had a selection marker similar to that in the plasmid pPgap-OsmY-Gluc1C, and the resultant transformant, SSY12, was grown in minimal medium containing cellobiose under the microaerobic condition. No significant cell growth or cellobiose utilization was observed during first 20 hr of cultivation (Fig. 5A). Cellobiose, however, was fermented beyond 20 hr and was completely utilized by 120 hr of cultivation. In addition to the cell growth, two major products were observed in the cell-free culture medium, i.e., ethanol and acetic acid, with the yields of 1.44 and 1.92 mM per mM cellobiose, respectively. We showed in our previous report that the ethanol yield and productivity using the SSY09(pZSack) strain were higher when the cells were grown in complex medium [25]. We therefore cultivated the SSY12 strain in LB medium containing cellobiose and observed an ethanol production with a yield of 3.2 mM per mM cellobiose (80% efficiency of biotransformation) (Fig. 5B). Production of significant amount of acetate indicated that the carbon present in the complex medium used for cultivation is also contributing to fermentative product formation.

### Fermentation of the biomass hydrolysate by the engineered strain

**Fig 6:**
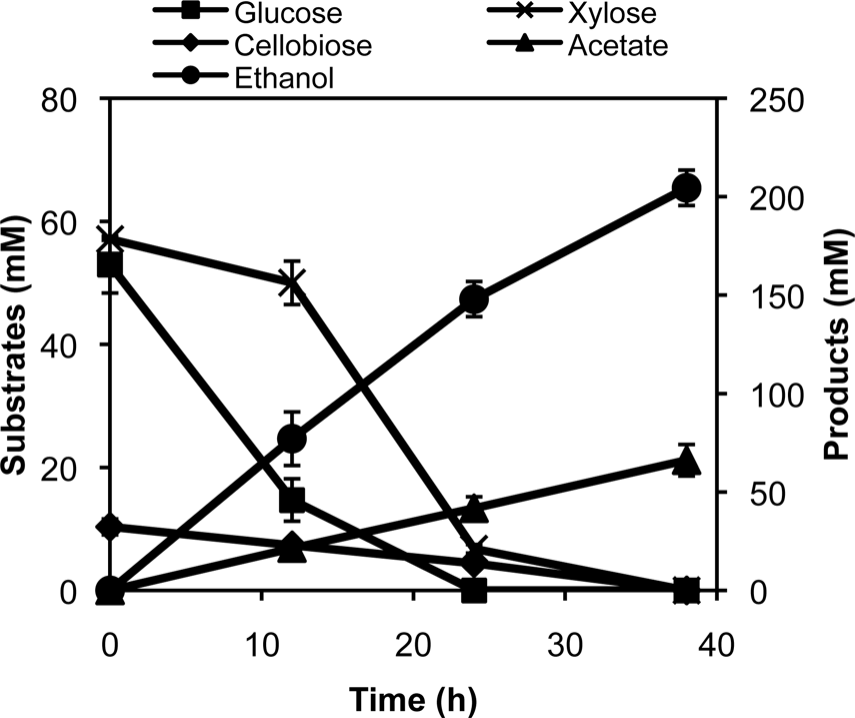
Time profiles of hydrolysate fermentation by the engineered strain. The engineered *E*. *coli* strain SSY12 bearing the plasmid pPgap-OsmY-Gluc1C was grown in LB+biomass hydrolysate medium under a microaerobic condition and the metabolites were monitored throughout the cultivation period. The data are presented as an average and standard deviation of two bioreactor batches.

The above studies confirmed that SSY12 secreted functional β-glucosidase into the extracellular medium, and this β-glucosidase hydrolyzed cellobiose into glucose units for further uptake and fermentation by the cells. We tested the fermentation of a wheat straw hydrolysate, which contained 53 mM glucose, 57 mM xylose and 10 mM cellobiose, by the SSY12 strain. All the three sugars were utilized by SSY12 in 38 hr and we obtained 204 mM of ethanol at the end of fermentation with 85% of efficiency of biotransformation (Fig. 6).

## Discussion

Industrial enzymes such as cellulases are used in variety of applications, the most important being the production of second-generation biofuels. Considering the low value of the end product the production cost for these enzymes need to be minimal. Therefore, use of expensive inducers and the inducible systems for producing these enzymes are undesirable. We screened the database to identify suitable constitutive promoters to derive the expression of cellulase enzymes both under aerobic and anaerobic condition. We considered only native constitutive promoters since the host RNA polymerase might not optimally recognize the heterologous promoters. We selected the promoters for the *gapA*, *ldhA*, and *pflB* genes since these promoters have been reported in previous studies to produce strong expression of the downstream genes in different strains [25, 26, 30]. Additionally, the corresponding genes had been shown to undergo a minimal change in expression level when Salmon et al. [31, 32] performed a transcriptomic study for a culture that was transformed from aerobic to microaerobic growth. Moreover, *gapA* transcription was found to be governed by multi-promoter region and interplay between different *gapA* promoters ensured high expression of GAPDH under various environmental conditions [33]. To test the relative strength of these promoters under both aerobic and anaerobic condition, we selected *lacZ* as a reporter gene and replaced its native promoter with the promoters for the *gapA*, *ldhA*, and *pflB* genes.

Initially, we tested the strength of these promoters by integrating them into the bacterial genome upstream of the *lacZ* gene since testing them in a plasmid system involves an extra element of variation, i.e., the plasmid copy number, which changes based on the origin of replication or the number of generations. While the *gapA* and *ldhA* promoters showed increased β-galactosidase activity, the *pflB* promoter resulted in decreased activity under the aerobic condition (Fig. 1). The low activity of β-galactosidase under the *pflB* promoter is expected because the product of the *pflB* gene, pyruvate-formate lyase, is known to be expressed and to function under anaerobic conditions [34]. Moreover, the transcriptomic data from a study conducted by Constantinidou *et al*. [35] indicated a 0.24-fold lower expression level of the *focA-pflB* operon under aerobic conditions compared with the expression under anaerobic conditions. The higher expression level for the *ldhA* promoter under the aerobic condition surprised us because its gene product is known to typically function under anaerobic conditions [36]. However, one study showed that the *ldhA* expression is highly pH dependent and at alkaline pH, the *ldhA* expression could be higher under aerobic conditions than under anaerobic conditions and vice versa [37]. Our cultivation condition in LB medium was slightly alkaline (~pH 7.4), and the result is thus consistent with the earlier observation. We further analyzed the promoter strength by cloning them upstream of *lacZ* in a ColE1 origin of replication-based plasmid. We found that the β-galactosidase activity in the plasmid-based system increased up to 95 fold and 400 fold in case of aerobic and anaerobic growth, respectively, as compared to the genomic integration-based system (Fig. 2). Placing the *lacZ* gene under the control of the *gapA* promoter resulted in the maximum expression of the β-galactosidase enzyme under both aerobic and anaerobic growth conditions and was therefore considered for further study.

One of the key bottlenecks in the production of lignocellulosic ethanol is the cost of hydrolytic enzymes. In our previous studies, we identified two important cellulolytic enzymes, β-1,4-endoglucanase and β-1,4-glucosidase, from a gut bacterium, demonstrated their overexpression, and characterized them biochemically [10]. We further demonstrated the construction of plasmids for the optimal secretion of these enzymes into the extracellular medium [24]. In that study, we used a T7 promoter-based expression system, pET28a(+), to secrete the enzymes. This expression system needed IPTG as an inducer, which is very expensive chemical that will drastically increase the cost of enzyme production. We therefore replaced the T7 promoter of this vector with the constitutive *gapA* promoter and analyzed the expression and secretion of enzymes. We found that the secretion of β-glucosidase was more efficient than that of endoglucanase, which could reflect specific properties of the proteins that affect their secretion from the *E*. *coli* host. We also observed a higher expression level of the enzymes under the control of the T7 promoter as compared to that of *gapA* promoter. This was not surprising since T7 promoter is one of the strongest inducible promoters known so far, and there is no competition for T7 RNA polymerase in the *E*. *coli* host [38, 39]. The expression of cellulases under the anaerobic growth condition at different time intervals, however, showed similar activity towards the later phase of growth. It will be beneficial to have a high specific activity of enzymes for the longer process under the anaerobic condition because ethanologenic *E*. *coli* may take longer than 24 hr to ferment sugars to ethanol [25].

It has often been observed that during the hydrolysis of plant biomass, a significant quantity of cellobiose remains unhydrolyzed due to the feedback inhibition of β-glucosidase that is present in the cellulolytic enzyme cocktail due to the glucose end product [40]. This cellobiose does not get fermented by the ethanol fermenting strain and becomes part of the waste stream. Researchers have followed different strategies to ferment cellobiose into ethanol. An ethanologenic KO11 strain was transformed to express the *Klebsiella oxytoca cel operon*, which works via the PTS system to hydrolyze the transported cellobiose [41, 42]. However, it expressed poorly in *E*. *coli*, and spontaneous mutations were promoted to yield better activity. A phosphorolytic mechanism for cellobiose assimilation has also been attempted in *E*. *coli* KO11 [43], where the cellobiose phosphorylase from *Saccharophagus* was expressed in the cytoplasm to utilize the cellobiose imported via endogenous lactose permease. There are other reports about the surface display of cellobiose-degrading enzymes [44, 45]. Most of these reports dealt with an inducible system with β-glucosidase that was either expressed intracellularly or displayed on the surface. In the present study, we report the constitutive expression of β-glucosidase that is secreted to the extracellular medium with the help of the OsmY tag in an ethanologenic strain engineered in our lab; this process facilitated the fermentation of cellobiose into ethanol. We also demonstrate that this recombinant strain fermented the biomass hydrolysate containing glucose, xylose and cellobiose and produced ethanol at a biotransformation efficiency of 85%. Therefore, an ethanologenic strain that constitutively secretes β-glucosidase is a promising strain for fermenting biomass hydrolysate.
